# Micropatterning biomineralization with immobilized mother of pearl proteins

**DOI:** 10.1038/s41598-021-81534-8

**Published:** 2021-01-25

**Authors:** Kristopher A. White, Vincent J. Cali, Ronke M. Olabisi

**Affiliations:** 1 Department of Biomedical Engineering, University of California—Irvine, Irvine, CA USA; 2 Department of Anatomy and Physiology, Queens College, City University of New York, Bayside, NY USA

**Keywords:** Biomaterials - proteins, Biomineralization

## Abstract

In response to the drawbacks of autograft donor-site morbidity and bone morphogenetic protein type 2 (BMP2) carcinogenesis and ectopic bone formation, there has been an increased research focus towards developing alternatives capable of achieving spatial control over bone formation. Here we show for the first time both osteogenic differentiation and mineralization (from solution or mediated by cells) occurring within predetermined microscopic patterns. Our results revealed that both PEGylated BMP2 and nacre proteins induced stem cell osteodifferentiation in microscopic patterns when these proteins were covalently bonded in patterns onto polyethylene glycol diacrylate (PEGDA) hydrogel substrates; however, only nacre proteins induced mineralization localized to the micropatterns. These findings have broad implications on the design and development of orthopedic biomaterials and drug delivery.

## Introduction

**Figure 1:**
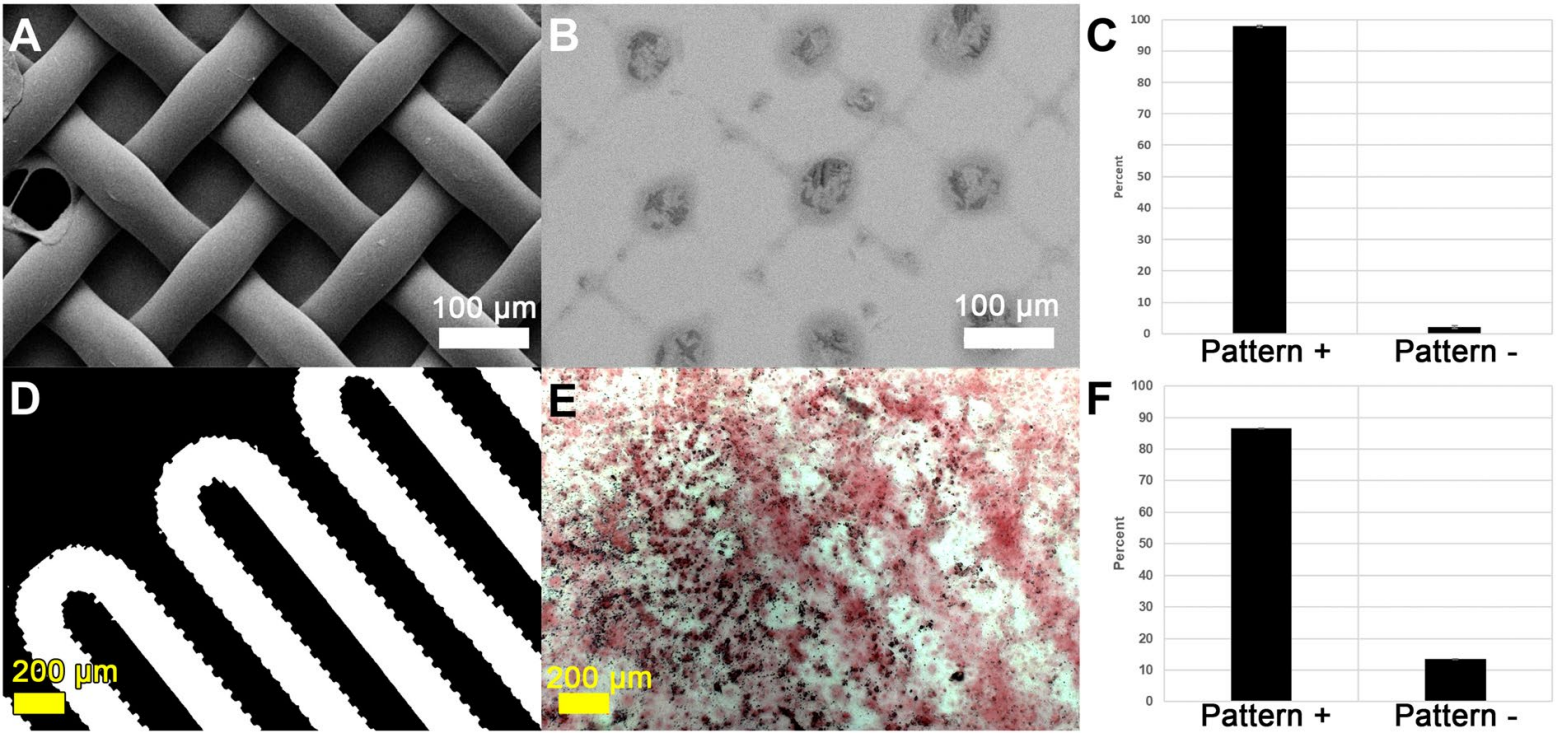
Patterned acellular (**A**–**C**) and osteoblast-directed mineralization (**D**–**F**) in response to PEG-WSM patterned substrates. (**A**,**D**) Photomasks, (**B**,**E**) resulting red-stained calcium mineralization and darker mineral nodules, (**C**,**F**) quantified mineralization that was contiguous with patterns.

As life expectancy increases worldwide, so does a need for bone tissue regeneration. Although the autograft is currently the clinical standard of care for iatrogenic bone repair, there remain significant complications and drawbacks such as morbidity at the harvest site and a finite amount available for harvest^1–3^. As an alternative, bone morphogenetic protein, type 2 (BMP2) is often delivered via collagen sponges; however, their relatively low affinity for BMP2 results in extremely high doses to achieve therapeutic bone formation^4^. Unsurprisingly, reports of complications due to these high BMP2 doses are on the rise, including the promotion of cancer and spine and/or airway impairments due to ectopic bone formation^5–9^. Despite a need to prevent such ectopic bone growth, spatiotemporal control over bone tissue formation as we report herein (Fig. 1) is relatively unreported in literature, particularly microspatial control^10^.

The formation of bone is a complex sequence of events involving both cell- and protein-mediated processes. In general, it involves the cell-guided development of a protein-based organic matrix followed by cellular deposition of osteoid, which crystallizes on the matrix in a manner controlled by the comprising proteins. Many proteins and growth factors play a role in bone formation. Some of these proteins stimulate cellular activity while others provide a template for the nucleation of bone mineral crystals^11–14^. BMP2, for instance, is known to induce differentiation in precursor cells toward the osteoblast lineage but has not been consistently shown to nucleate calcium from solution^15^. Bone sialoprotein (BSP), on the other hand, has been shown to readily nucleate and self-assemble apatite (calcium phosphate) crystals in solution^16^. In this way, certain proteins responsible for biomineralizing bone have parallel roles to proteins that biomineralize seashell. Seashell consists entirely of calcium carbonate: the rough outer prismatic layer is composed of the polymorph calcite, while the polymorph aragonite comprises the inner lustrous nacreous layer (also known as mother of pearl). The water-soluble matrix (WSM) of nacre has been shown to nucleate and self-assemble aragonite crystals in solution as well as to play a significant role in an osteogenic response in mammalian cells^17,18^. An isoform of n16, a nacre-derived protein, self-assembles protein oligomers that dimensionally limit and organize calcium carbonate mineral deposits, but does not appear to induce osteodifferentiation nor osteogenesis^19^. The peptide, n16N, derived from the n16 protein, has also been shown to nucleate calcium from solution^19^, but prior to the work herein had not been shown to exert an osteogenic effect on mammalian cells. In essence, some nacre proteins could either nucleate calcium or initiate osteogenesis, yet any link between these two processes had not been elucidated. Likewise, in bone biomineralization, although headway has been made in determining which bone-derived proteins are “osteonucleators” and which are osteoinductors, little data exists to elucidate any relationship between acellular mineralization and osteogenic activity, and few studies have directly compared the two processes^10^. This relationship between cell- and protein-mediated processes may further the understanding of microspatial control of bone tissue formation.

Approaches to microspatial control of bone formation has generally involved a tissue engineering approach, incorporating known osteogenic substances into biomaterials^20,21^. For instance, ground seashell and bone-derived proteins have been incorporated into polyethylene glycol (PEG) hydrogels to direct bone formation in bulk or in macroscopic patterns^22^. PEG is a hydrophilic, bioinert polymer that is easily modified to fit numerous biomedical engineering applications in both tissue engineering and drug delivery. In particular, PEG can be covalently attached to therapeutics, growth factors, proteins, peptides, and other small molecules, and these in turn can be incorporated into various substrates ranging from hydrogel networks to glass surfaces^23,24^. Incorporation of these “PEGylated” biofactors into a larger PEG hydrogel “functionalizes” an otherwise bioinert hydrogel^23,25^. Such functionalized PEG hydrogel networks have been shown to exhibit high affinities for therapeutics, which enables control of the presentation and release of the therapeutics, both in vitro and in vivo^22,25^. Although moieties covalently bound to PEG have been used to guide angiogenesis and cell-mediated biomaterial degradation, prior to this work spatial control over bone formation using PEGylated molecules had only been accomplished by restricting osteoprogenitor cell access to attachment sites like RGDS rather than by PEGylating osteogenic proteins in specified patterns^26–29^. In short, attachment sites were patterned, not mineralization sites. Our approach to directing bone formation has been to pattern mineralization sites, thereby combining a biomineralization strategy with tissue engineering. Here, we immobilize bone- and nacre-derived proteins in patterns on PEG hydrogels to for the first time achieve control over the location and onset of acellular mineralization and osteoprogenitor cell-mediated biomineralization of calcium in vitro. Because pilot studies confirmed others’ findings that the water soluble component of nacre but not the urea soluble nor EDTA soluble components were capable of inducing osteogenic activity in mammalian osteoblasts^17,30,31^, we used only the water soluble matrix from nacre in our studies. Additionally, we demonstrate for the first time that the mineral nucleation ability of un-PEGylated and PEGylated bone and seashell proteins may explain their osteogenic abilities.

## Results

### Bone and nacre driven acellular mineralization

**Figure 2:**
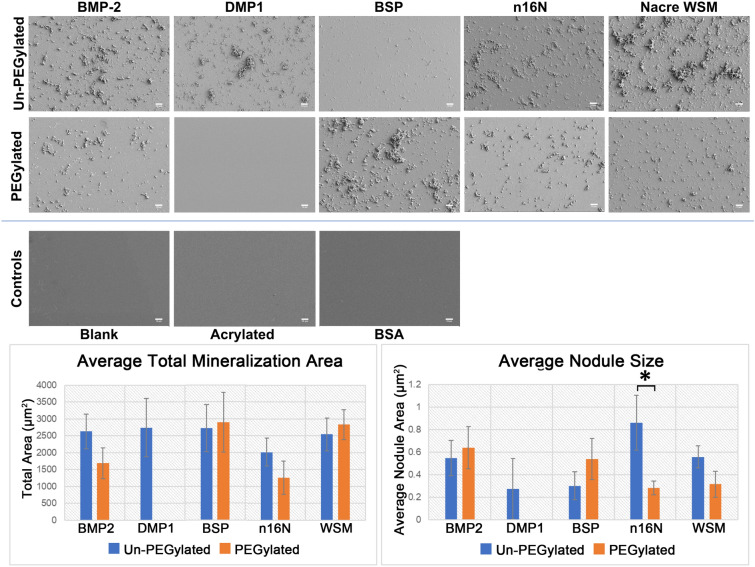
FE-SEM images of glass slides coated in PEGylated and non-PEGylated proteins or peptides following mineralization assays. Slides were acrylated and PEG-proteins were covalently bound or cleaned slides were adsorbed with un-PEGylated proteins. Controls, clean blank slides, acrylated slides, and slides with adsorbed BSA did not induce mineralization and are therefore featureless. Scale bars are 10 μm. Error bars show standard deviation. Asterisks denote significant difference between indicated samples (*p* < 0.05). *Note* No mineralization was observed in PEGylated DMP1 samples.

Un-PEGylated proteins derived from bone or nacre were successfully adsorbed to cleaned glass slides while PEGylated bone and nacre proteins were successfully covalently bound to acrylated glass slides. Field emission-scanning electron microscopy (FESEM) images of the protein-adsorbed slides post-nucleation showed surface features consistent with calcium phosphate mineralization following immersion in a calcium phosphate bath (Figs. 1, 2). Additionally, increased mineralization was observed for all proteins compared to negative control slides. PEGylated BMP2 (PEG-BMP2), PEG-BSP, and PEG-WSM also appeared to induce acellular mineralization. In contrast, no acellular mineralization occurred in any PEG-DMP1 samples. FE-SEM images of PEG-WSM taken before mineralization assays indicated that acrylation and protein conjugation caused no change in surface features on the glass slides. Surface-adsorbed bovine serum albumin (BSA) also caused no visible changes, suggesting that any changes in surface characteristics were due to mineralization induced only by proteins capable of acting as mineral nucleators.

NIH ImageJ particle analysis revealed subtle differences between slides (Fig. 2 graphs), with mineralization reported as the total area occupied by mineral crystals per field of view. The PEG-BSP slides showed the highest average total mineralization area (2890.4 ± 882.7 µm^2^), but all un-PEGylated samples were comparable to one another. PEGylated samples, however, showed greater variance. No mineralization was observed in PEG-DMP1 samples. PEG-BMP2 (1685.2 ± 451.2 µm^2^) and PEG-n16N (1256.8 ± 486.2 µm^2^) showed decreased mineralization compared to their non-PEGylated counterparts (2631.6 ± 513.0 and 2012.8 ± 411.1 µm^2^, respectively). Both PEG-BSP (2890.4 ± 882.7 µm^2^) and PEG-WSM (2833.6 ± 443.5 µm^2^) samples showed similar or slightly increased mineralization area compared to BSP (2715.1 ± 697.5 µm^2^) and WSM (2542.4 ± 487.7 µm^2^), respectively. PEGylation and immobilization appeared to increase nodule size in PEG-BMP2 (0.64 ± 0.19 µm^2^) and PEG-BSP (0.54 ± 0.18 µm^2^) samples but decreased average nodule size in PEG-WSM (0.32 ± 0.11 µm^2^) and PEG-n16N (0.28 ± 0.06 µm^2^) samples, with PEG-n16N resulting in significantly decreased average nodule size compared to non-PEGylated n16N (0.86 ± 0.24 µm^2^).

#### Submicron mineralization differences

**Figure 3:**
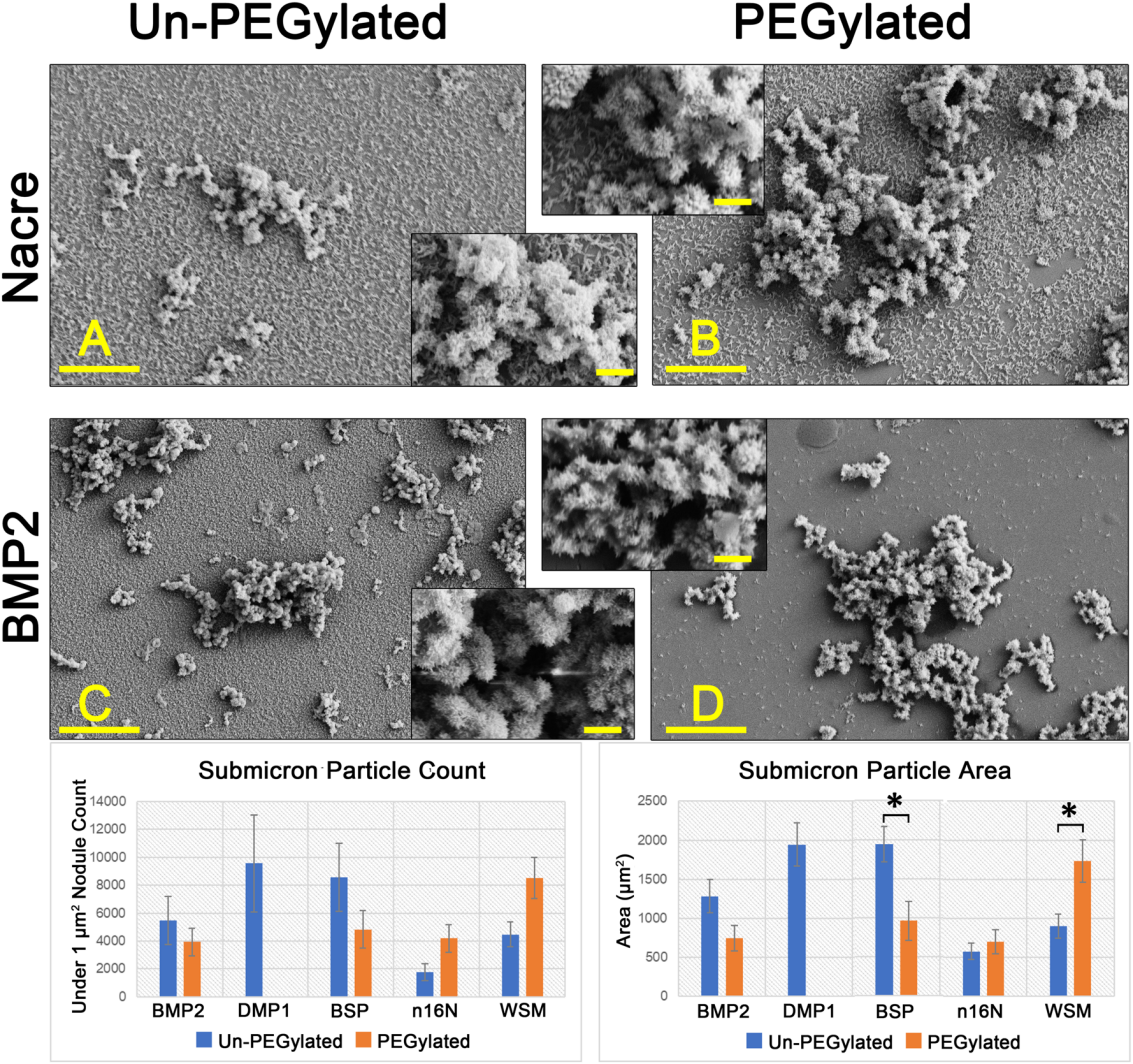
FE-SEM images at ×10,000 magnification of slides coated with (**A**) WSM, (**B**) PEG-WSM, (**C**) BMP2, and (**D**) PEG-BMP2. Mineralization features below 1 µm can be seen as a “dusting of snow” in both non-PEGylated proteins BMP2 and WSM. PEGylation inhibits this type of mineralization in BMP2 but not in WSM as they are present in the PEG-WSM slide but not on the PEG-BMP2 slide. All four samples revealed crystals with an acicular habit. Main scale bars are 2 μm; inset scale bars are 1 μm. Error bars show standard deviation. Asterisks denote significant difference between indicated samples (*p* < 0.05). *Note* No mineralization was observed in PEGylated DMP1 samples.

Higher magnification revealed submicron features on the PEG-WSM slides (Fig. 3). No such features were present on the PEG-BMP2 slides (Fig. 3D). When thresholding was bound at a maximum particle size of 1 µm^2^, a particle count confirmed this observation, as did an assessment of the total area of submicron particles observed. Further analysis revealed that PEGylation of WSM and n16N increases the occurrence of sub-micron features whereas PEGylation of the other proteins resulted in the decreased presence of these features relative to their non-PEGylated counterparts.

#### Patterned acellular mineralization

**Figure 4:**
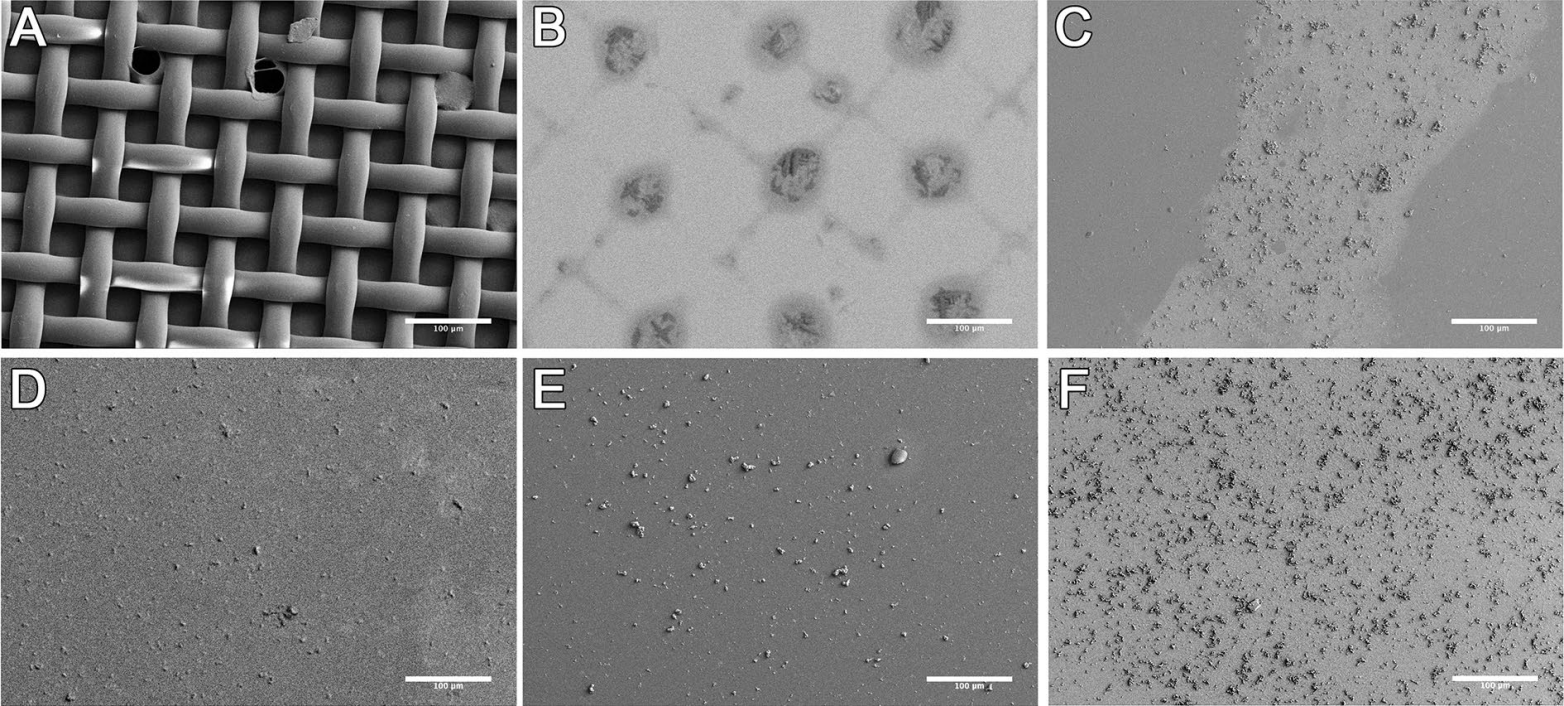
FE-SEM micrographs of mineralization on glass slides patterned with PEGylated proteins. (**A**) A nylon mesh photomask was used to pattern (**B**) PEGylated Nacre WSM, (**C**) BMP2, (**D**) DMP-1, (**E**) BSP, and (**F**) n16N. Scale bars are 100 µm.

Patterned PEG-WSM resulted in the development of crosshatched areas of mineralization from solution (Figs. 1, 4), which corresponded to a projection of the photomask onto the slide. After calculating the size of the projection, mineralization associated with the photomask pattern was determined to be 97.87% (Fig. 1). Patterned mineralization consistent with the pattern of the photomask was not observed in any of the other protein samples (Fig. 4C–F).

### Bone and nacre driven cellular mineralization

**Figure 5:**
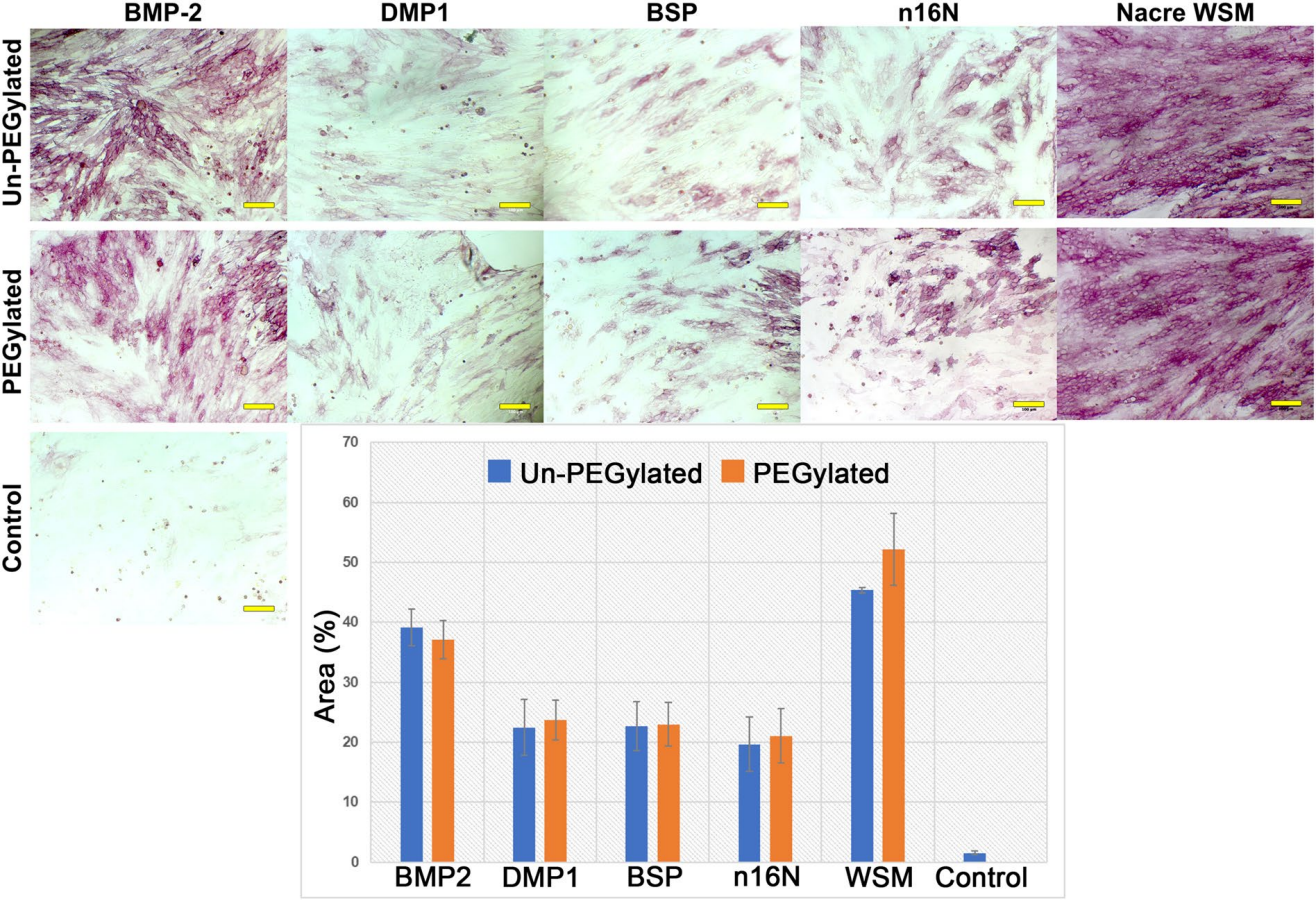
ALP-stained W-20-17 cells seeded onto PEGDA hydrogels incorporating PEGylated or non-PEGylated osteogenic proteins and peptides. Control cells were seeded on PEGDA hydrogels containing no proteins. ALP expression in W-20-17 cells was thresholded, then quantified as percent area covered. Error bars indicate standard deviation. Scale bars are 100 µm. Images were uniformly adjusted for brightness and contrast.

After 4 days of growth on PEGDA hydrogels with PEGylated or non-PEGylated bone or nacre derived proteins, W-20-17 stem cells stained positive for alkaline phosphatase (ALP) expression, a marker of osteodifferentiation (Fig. 5). All samples showed significantly increased ALP activity compared to control gels without protein incorporation. ALP expression, reported as percentage of area, appeared to be highest in BMP2 and WSM samples. All samples appeared to retain the ability to induce osteodifferentiation in W-20-17 cell monolayers when PEGylated. When all images were thresholded against a standard background, the highest area percentage of pigmentation was present in the WSM (45.37 ± 0.47%) images followed by BMP2 (39.17 ± 3.04%) (Fig. 5 graphs). PEGylation did not significantly affect osteodifferentiation in any protein or peptide samples.

#### Mineralization in 2D culture on PEGDA hydrogels

**Figure 6:**
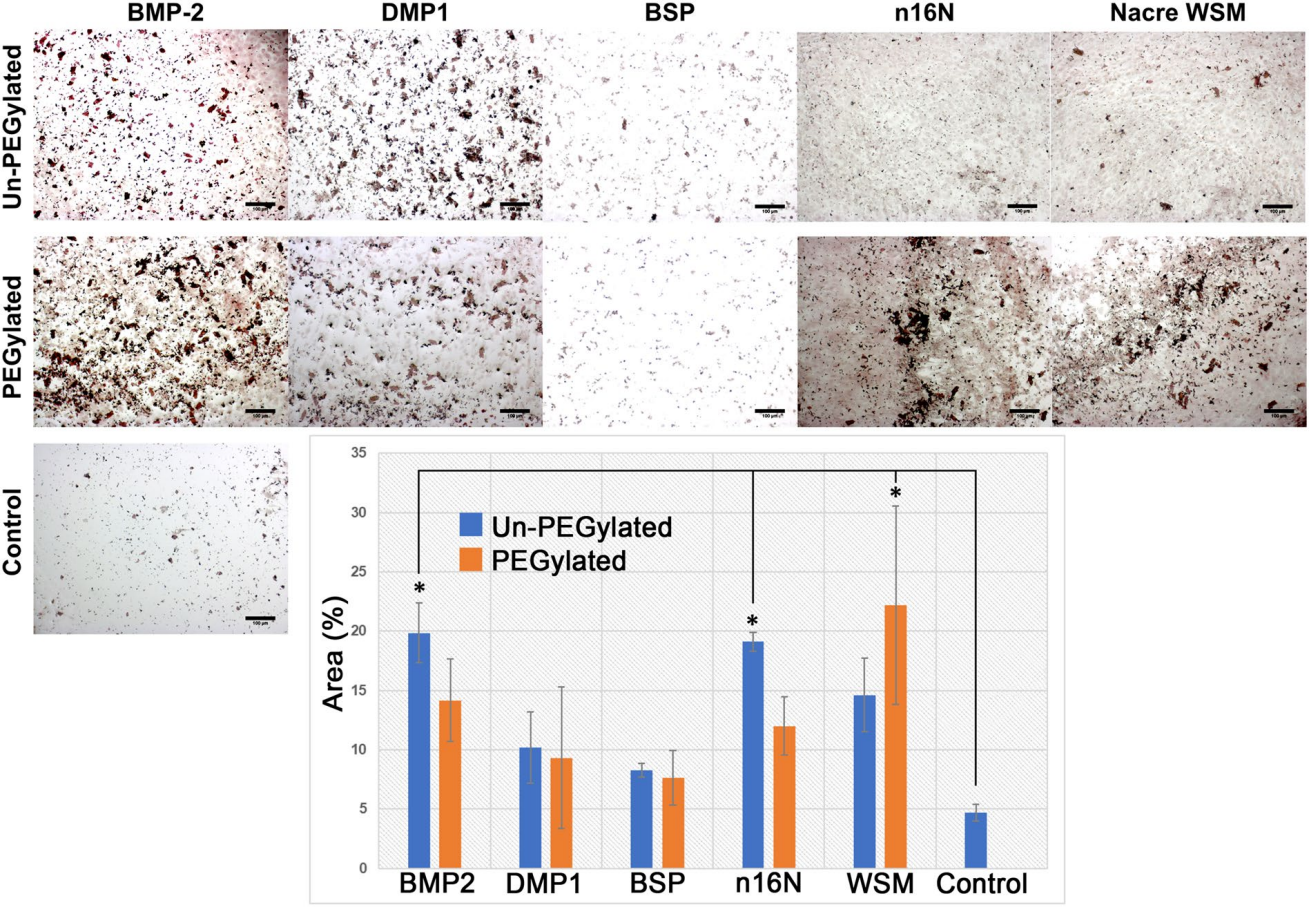
MC3T3-E1 cells on PEGDA hydrogels incorporating PEGylated and non-PEGylated proteins. Cells stained with Alizarin red S to visualize calcium, which is stained red. Error bars represent standard deviation. Scale bars are 100 µm. Asterisks denote significant difference between sample and protein negative control (*p* < 0.05). Images were uniformly adjusted for brightness and contrast.

After 12 days of culture on PEGDA hydrogels, MC3T3-E1 preosteoblast cells stained with Alizarin Red S (ARS) stain, which stains calcium deposits red, revealed differing levels of calcium mineralization on hydrogels incorporating PEGylated or un-PEGylated proteins (Fig. 6). PEGylation appeared to negatively impact mineralization area in PEG-BMP2 (14.16 ± 3.48%) and PEG-n16N (12.00 ± 2.45%), though this decline was not significant when compared to their non-PEGylated counterparts (19.85 ± 2.52 and 19.11 ± 0.81%, respectively). Although the increased mineralization of PEG-WSM (22.17 ± 8.37%) over WSM (14.63 ± 3.08%) was not significant, PEG-WSM is the only PEGylated protein sample to show significantly increased mineralization compared to protein-negative controls (4.69 ± 0.72%). Among non-PEGylated proteins, only BMP2 and n16N showed significantly increased mineralization compared to negative controls.

#### Mineralization in 3D culture in PEGDA hydrogel microspheres

**Figure 7:**
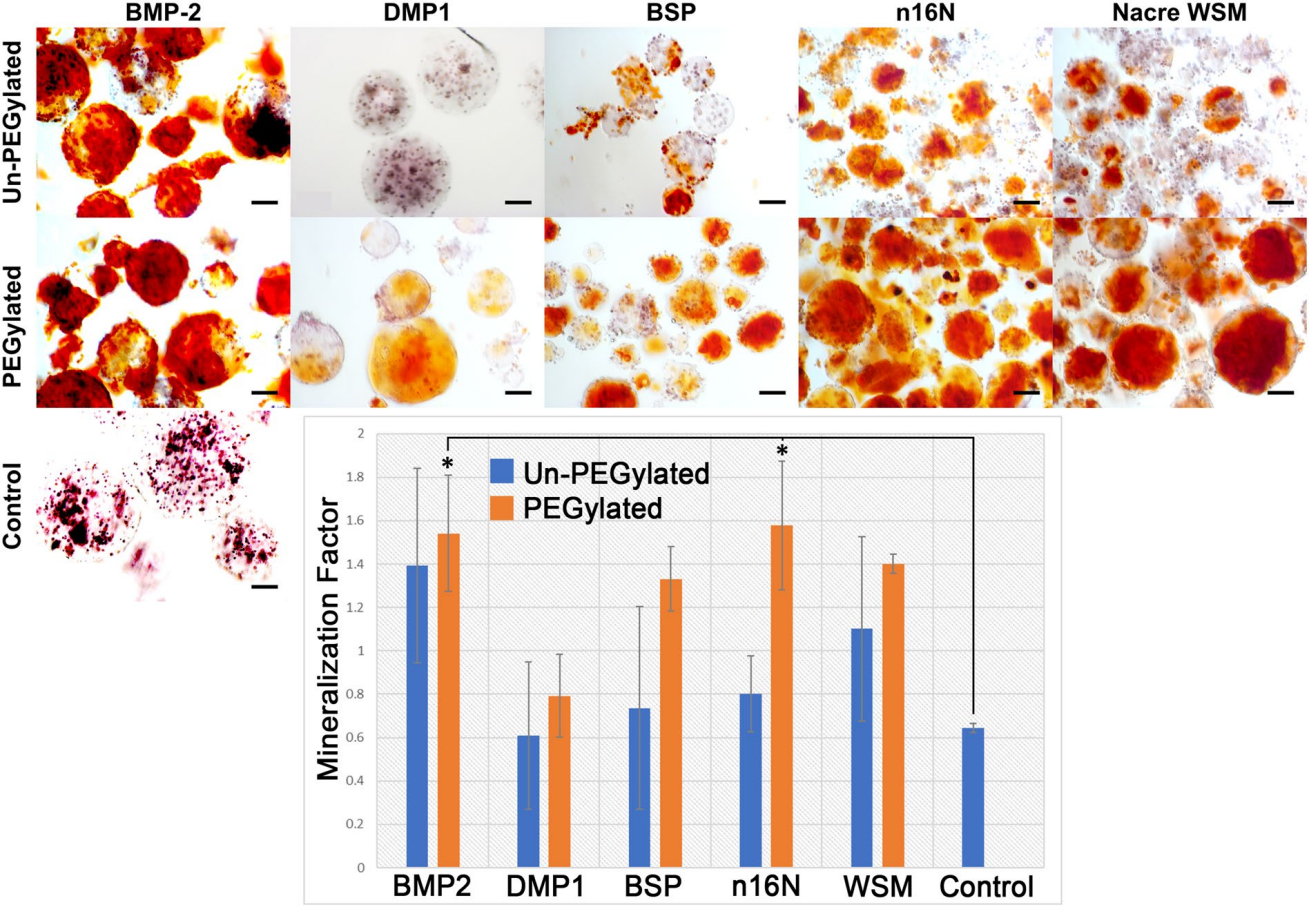
Microencapsulated MC3T3-E1 cells stained with ARS. Scale bars are 100 µm. Error bars represent standard deviation. Asterisks denote significant difference compared to protein-negative controls (*p* < 0.05).

Microsphere encapsulation permitted 3D culture with sufficient nutrient/waste diffusion in addition to easy visualization of calcium stains. Day 21 ARS staining of microsphere-encapsulated MC3T3-E1 cells containing PEGylated or non-PEGylated proteins and peptides showed elevated mineralization when compared to hydrogel microspheres prepared without added proteins (Fig. 7). Cellular mineralization appeared to increase with immobilization for all proteins; however, no significant difference due to PEGylation was observed. When quantified, PEG-BMP2 (1.54 ± 0.27) and PEG-n16N (1.58 ± 0.04) showed significant differences in mineralization compared to the negative control.

#### Patterned cellular osteogenic response

**Figure 8:**
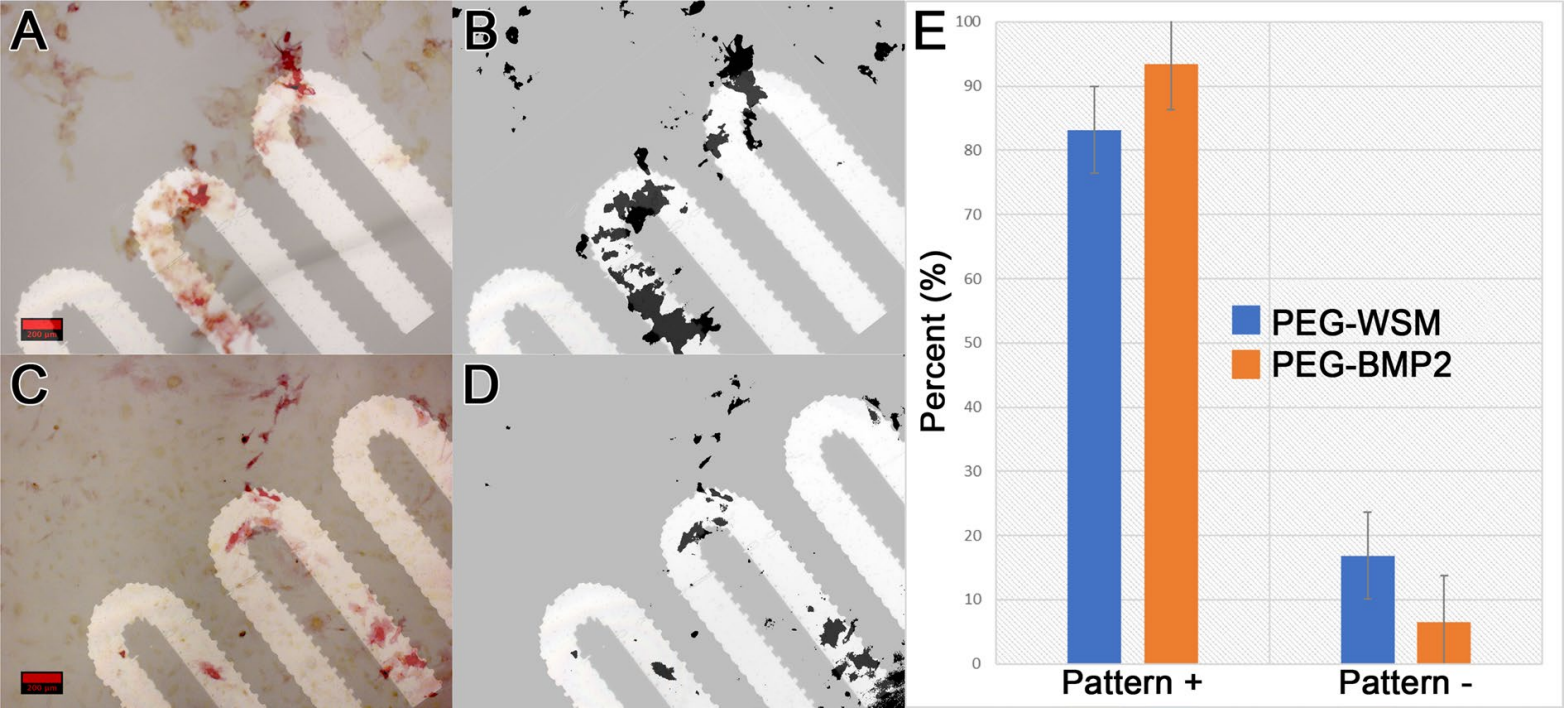
ALP-stained images of PEGDA hydrogels incorporating patterned PEGylated WSM (**A**) and BMP2 (**C**) were thresholded for intensity and overlaid with pattern (**B**,**D**). Mineralization associated with the overlaid patterns were quantified (**D**). Graph indicates percentage of stained cells associated with patterned PEGylated WSM. Scale bars are 100 µm. Error bars represent standard deviation.

**Figure 9:**
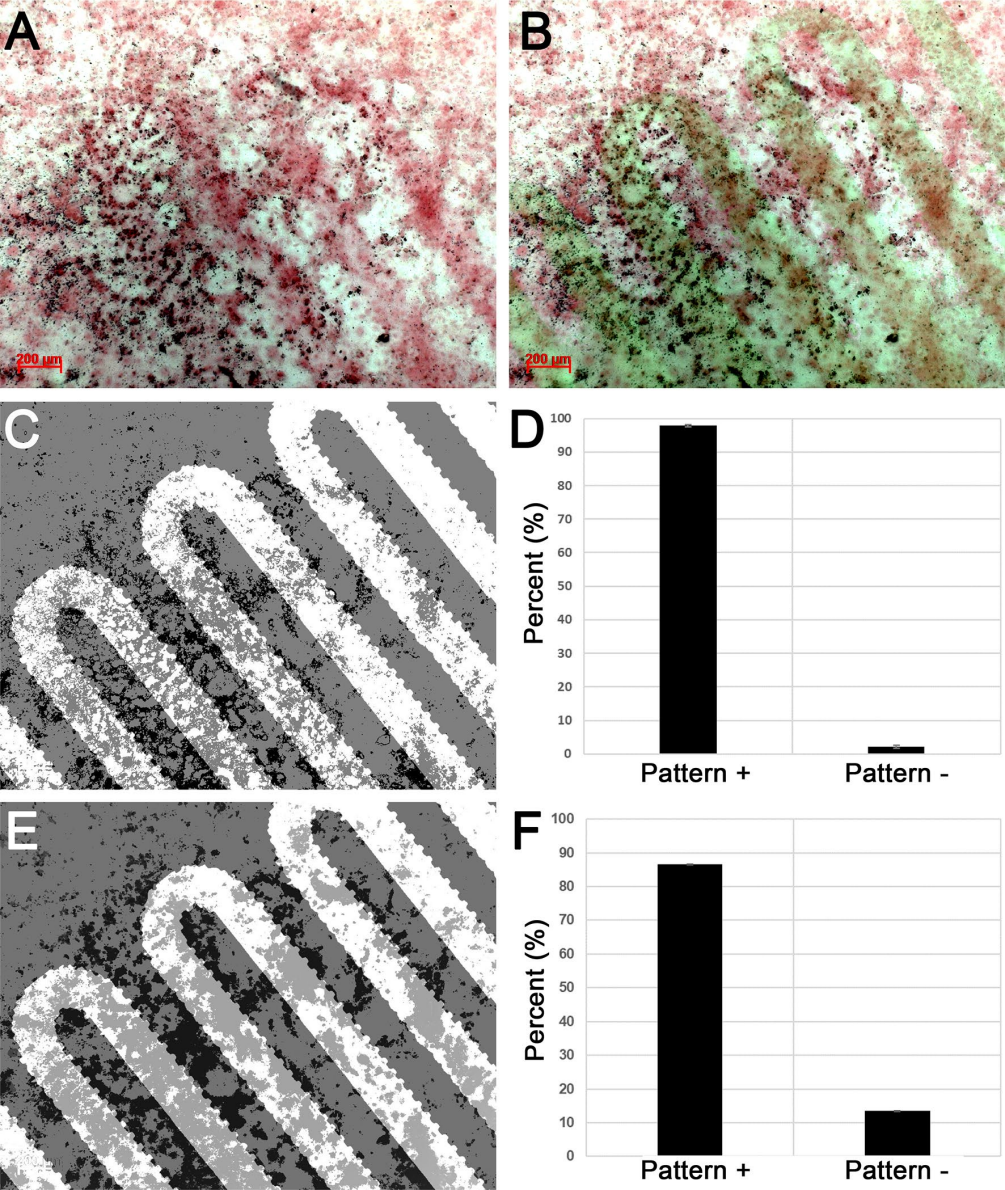
Quantitated mineralization. (**A**) Day 12 MC3T3-E1 cells seeded onto PEG hydrogels patterned with PEG-WSM proteins and stained with Alizarin red S. (**B**) ARS-stained cells from (**A**) overlaid with fluorescent pattern. (**C**). Thresholded binary image of mineral nodules (appear dark in **A**) overlaid with photomask pattern. (**D**) Mineral nodules contiguous with the overlaid pattern was quantified. (**E**) Thresholded binary image of red calcium stain in the monolayer overlaid with photomask pattern. (**F**) Quantified red calcium stain contiguous with the photomask pattern.

When PEG hydrogels patterned with covalently bound osteogenic proteins were seeded with either W-20-17 mesenchymal stem cells (Fig. 8 and Figure S4) or MC3T3-E1 preosteoblast cells (Fig. 9 and Figure S5), PEG-WSM and PEG-BMP2 showed patterned differentiation. ALP expression correlated to the pattern was determined to be 83.17% and 93.48% in hydrogels patterned with PEGylated WSM and BMP2, respectively (Fig. 8). Positive ARS stain and mineral nodule formation were observed in patterns in MC3T3 cells after 12 days when cultured on hydrogels patterned with nacre WSM (Fig. 9), but not in patterns with other pegylated proteins. When analyzing images from pilot data^31,32^, mineralization stained with von Kossa was calculated to be 90.20% within or connected to patterns. Mineralization in this study associated with the PEG-WSM patterns was calculated to 86.54 ± 2.58% (red ARS stain) and 84.62 ± 0.36% (darker mineral nodules).

## Discussion

Clinically approved bone regeneration techniques exhibit poor spatial control over osteogenesis. Our approach to this problem has been to immobilize known and suspected nucleators of calcium, in essence borrowing from the high spatial control that occurs in biomineralization to exert spatial control in bone tissue engineering. Our findings strongly suggest that proteins capable of nucleating calcium from solution are osteogenic, while proteins incapable of nucleating calcium—such as bovine serum albumin—are not osteogenic. Our study may offer insights into which osteogenic molecules are the best targets for spatially controlling bone formation.

For the first time, this work demonstrates that patterned nacre WSM is capable of microspatial control over osteogenic differentiation and mineralization. Furthermore, this study suggests that proteins capable of nucleating calcium from solution in patterns may also be capable of mediating osteogenic activity in patterns. Acellular mineralization studies revealed that PEGylating nacre proteins increased submicron mineralization while PEGylating bone proteins decreased submicron mineralization. In particular, PEG-WSM proteins increased mineralization on the sub-micron scale more than all other PEGylated proteins. Because PEG-WSM was the only protein capable of controlling both acellular and cell-mediated mineralization, the sub-micron features observed may be key to achieving this control. This increased presence of sub-micron mineralization on slides with immobilized PEG-WSM compared to slides with PEG-BMP2 may be the nucleation sites required for highly ordered mineral deposition, potentially elucidating PEG-WSM’s apparent capacity for microspatial control. The submicron mineralization observed may indicate a relationship between nacre WSM’s ability to organize calcium mineralization and its ability to spatially direct cell-mediated mineralization. For instance, He. et al. have demonstrated that surface-adsorbed DMP-1 nucleates calcium phosphate from solution^33^. In vivo, DMP-1 binds self-assembled collagen and mediates the ordered deposition of nanocrystalline hydroxyapatite^34^. This acellular nucleation of minerals may be an important first step in the cell-mediated calcification process^34^. Attaching PEG molecules to DMP-1 completely inhibited DMP-1’s ability to nucleate calcium phosphate from solution. Although attaching PEG molecules to BMP and BSP did not eliminate their nucleation ability, it altered it. Nacre WSM was the only protein that appeared to gain nucleation capacity when bound to PEG, potentially paving the way to use PEG-bound nacre WSM to mediate the ordered deposition of nanocrystalline calcium in the same way collagen-bound DMP-1 controls nanocrystalline hydroxyapatite. A significant hurdle in exerting microscale control over osteogenesis has been effectively immobilizing therapeutics, thereby preventing their escape from treatment sites. Bulk immobilization of therapeutics has been demonstrated; for instance, VEGF shows a higher affinity and slower release profile in acidic gelatin substrates while BMP2 performed more favorably in basic gelatin substrates^35–37^. Affinity also operates on a relative scale wherein release profiles can be accelerated in vivo via displacement by other factors with a higher affinity for the substrate. Although these methods show some success, affinity approaches introduce variables as the release of the therapeutic molecules can vary between experiments. Here, we covalently immobilized the growth factors via a PEG linker to mediate or eliminate these variables. This approach led to the successful mineralization of calcium in predetermined microscopic patterns, both from solution and as directed by osteoprogenitor cells.

In addition, although n16N had been shown to nucleate aragonite, this work is the first to show its osteogenic properties, both prior to and followingPEGylation. Furthermore, we demonstrate that several bone- and nacre-derived proteins retain their bioactivity and ability to nucleate minerals when PEGylated despite their inability to control osteogenesis within prescribed patterns. BMP2, n16N, and PEG-WSM showed increased cellular mineralization over the protein-negative control. These results confirm previous findings that BMP2 and nacre WSM induce osteogenesis and mineralization in vitro^15,17^, while BSP and DMP-1 had a less pronounced effect^13,38^. When PEGylated, all proteins in this study appeared to retain their osteogenic potential in three-dimensional microspheres, despite PEG-DPMP-1 losing its ability to initiate acellular mineralization. This offers promise that other osteogenic proteins may be propitious options in the search for BMP2 alternatives; even PEG-BMP2, when bound to a biomaterial, might avoid ectopic bone growth. Furthermore, the osteogenic potential of n16N, both before and after PEGylation, may indicate that other osteogenic peptides—which are often cheaper than whole proteins—can be covalently bound to substrates to direct bone growth.

Although this study demonstrated microspatial control of bone formation, there remain limitations. It is possible that factor retention within the hydrogel matrix led to increased cellular exposure in the 3D microspheres. It is also possible that these hydrogel microspheres trapped cellular products, such as collagen or secreted growth factors, leading to increased activity within a given microsphere. This may explain the marked difference in osteogenic activity between the 2D and 3D cultures. Incorporating PEGylated growth factors within the bulk of 2D scaffolds may have resulted in the availability of only a fraction of the proteins near the cell-seeded surface for the duration of the experiment. Additionally, entrapped non-PEGylated proteins may have become available to the cells as the proteins eventually migrated out to the surfaces of the gels, where the cells were seeded. This gradual replenishment of growth factors may have mitigated any difference between the observed cellular effects between PEGylated and non-PEGylated proteins in the 2D culture. Despite these limitations, PEGylated nacre WSM showed the ability to direct both acellular and cell-mediated mineralization in patterns. Exploiting this ability may lead to orthopedic biomaterials capable of spatially controlling bone formation and eliminating ectopic bone growth.

## Materials and methods

### Extraction of nacre water-soluble matrix

The shells of the giant oyster Pinctada maxima were obtained from the Philippines. The WSM was extracted using modified non-decalcifying procedures described elsewhere^17,30^. The inner nacreous layer of the shells was stripped from the outer calcite layer using a tungsten carbide drill bit. Because sterilization procedures that either heated nacre powders or filtered nacre extracts showed no difference in osteogenic activity^17,30,39^, the resulting powder (50 g) was sterilized via dry heat (70 °C, 1 h) to eliminate contamination and preserve proteins that might be lost to adsorption during filtration. The sterile powder was then immediately placed into a sterile container with 100 mL cold ultrapure water (~ 18 MΩ-cm) and stirred under refrigeration for four days. Afterward, the slurry was transferred into 50 mL conical centrifuge tubes and centrifuged at 3700*g* for 20 min. The supernatant was then transferred into clean conical tubes and lyophilized until dry and stored at − 20 °C. The resulting material was then weighed and used as-is, designated as nacre WSM.

### Synthesis of PEGylated proteins and peptides

Nacre proteins were extracted as described above. BMP2 (Life Technologies, PHC7141), BSP, (R&D Systems, 4014-SP-050),  DMP-1 (Life Technologies, 11929H08H50), and the synthetic cell adherent peptide RGDS (MedChemExpress, HY12290) were purchased and used as received. The synthesis of the aragonite-nucleating nacre-derived peptide^19^, n16N (Biomatik, custom order), was outsourced and used as received. Proteins were PEGylated by combining them with Acrylate-PEG-succinimidyl valerate (SVA) (Laysan Bio, Inc.) in HEPES buffered saline (HBS, pH 8.5) at a 20:1 PEG-SVA:protein molar ratio for BMP2, a 16:1 molar ratio for BSP, a 21:1 molar ratio for DMP-1, a 5:1 molar ratio for n16N, and a 100:1 PEG-SVA:nacre WSM weight ratio. The molar stoichiometric amounts are relative to the number of primary amine residues in the protein sequence. The ratios for WSM and n16N were selected to ensure excess PEG-SVA would be present for the reaction.

### Confirmation of protein conjugation

Protein-PEG conjugation was confirmed using the ninhydrin assay for free amines. Ninhydrin reacts with free amines to form a purple dye called Ruhemann’s purple. This purple reaction product is used in a colorimetric assay to measure the total available free amines in a given peptide or protein sample. After reacting the proteins and peptides with acrylate-PEG-SVA, samples were lyophilized until dry and reconstituted in 100 µL phosphate buffered saline (PBS, pH 7.4). The solutions were then separately added to 100 µL sodium citrate buffer (pH 5.0) and 200 µL ninhydrin solution (2%) in low protein binding microcentrifuge tubes. The tubes were heated to > 95 °C for 15 min to facilitate the reaction. The absorbance was then read at 570 nm. Standard curves for all proteins and peptides were prepared using known concentrations of each. The conjugation efficiency was reported as the quotient of occupied amines post-conjugation to available amines pre-conjugation.

### Synthesis of surface-immobilized protein substrate

Nucleation capacity was tested on glass slides coated with proteins. The nucleation protocol was adapted from He et al.^33^ The glass slides were cleaned in an acidic mixture of concentrated hydrochloric acid (HCl, 22.5%) and nitric acid (HNO_3_, 7.5%) to remove any residual biological contaminates and to condition the slides for subsequent adsorption steps. The slides were placed into the acid mixture and subjected to sonication (50 kHz) for 1 h at 60 °C. The slides were then washed in ultrapure water five times. Each wash lasted 1 min under sonication. The washed slides were placed into a HEPA-filtered incubator overnight to dry. The slides were then coated with proteins by placing 50 µL protein solution (0.2 mg/mL in ultrapure water) onto the surface of the glass slides. The slides were again allowed to dry overnight for protein deposition, after which they were washed twice in cold ultrapure water to remove excess and unadsorbed proteins.

PEGylated proteins were immobilized on the glass slides via a photoconjugation reaction. Slides were cleaned in concentrated HCl/HNO_3_ as described above. The clean slides were then acrylated using 3-(trimethoxysilyl)propyl acrylate to permit conjugation of PEGylated proteins. 3-(trimethoxysilyl)propyl acrylate was dissolved in chloroform at 0.1% by volume. The slides were coated with 50 µL of the acrylate solution pipetted onto the surface of the glass slide. Care was taken to completely cover the surface. The slides were then dried overnight in a HEPA-filtered environment. Once dry, a thin acrylate film could be seen on the surface of the slide. The slides were washed twice in cold ultrapure water to remove excess acrylate. Once washed and dried, no film was visible to the naked eye on the glass surface. Further, the absence of film was confirmed via light and electron microscopy, indicating that only adsorbed acrylate groups were present and that a poly acrylated surface did not form. After the acrylated slides were dry, they were coated with 50 µL PEGylated protein solution (2 mg protein equivalent per mL ultrapure water), taking care to cover the entire slide to facilitate homogenous distribution of protein. The slides were exposed to white light for 1 min to facilitate surface conjugation via photo-initiated acrylate coupling between the PEGylated proteins and the acrylated slide surface. The protein-conjugated slides were then washed twice with cold ultrapure water to remove unbound proteins.

### Acellular mineralization

The slides containing surface-adsorbed and surface-conjugated proteins were placed into the center of a modified horizontal electrophoresis chamber such that a thin film of buffer would form over the slides once the chamber was filled (Figure S2). The anode chamber was filled with a calcium buffer (165 mM NaCl, 10 mM HEPES, 2.5 mM CaCl_2_, pH 7.4) and the cathode chamber was filled with phosphate buffer (165 mM NaCl, 10 mM HEPES, 1 mM KH_2_PO_4_, pH 7.4). The thin film of buffer allowed the ions to move freely across the surface of the slides, where the proteins could facilitate Ca/P binding. An external 10 mA current was applied to prevent non-specific mineral nucleation. The buffer was changed twice per day to maintain pH and ion concentration. After 3 days, the slides were removed from the buffer and washed once in cold water to remove any residual buffer. The slides were then dried under vacuum and sputter coated with gold (10 nm) before imaging on a Zeiss Sigma Field Emission SEM equipped with an Oxford INCA PentaFETx3 EDS system.

The resulting FE-SEM images were analyzed via ImageJ software. The images were first thresholded to remove the background and converted to black and white (Figure S3). The images were then analyzed for particle sizes > 1 µm. The resulting particle analysis was then used to calculate the total mineralized area of each slide and the average particle size.

### Acellular patterning of mineralization

PEGylated proteins were patterned onto the surface of acrylated slides via photolithography. As previously described, after the acrylated slides were dry, they were coated with 50 µL PEGylated protein solution (2 mg protein equivalent per mL ultrapure water), taking care to cover the entire slide to facilitate homogenous distribution of protein. A crosshatched nylon photomask was then placed over the top of the solution such that only openings in the mesh were exposed to focused white light for 1 min. For acellular patterning, the nylon meshes offered higher resolution than the photomasks used for hydrogel patterning, permitting visualization of mineralization with electron microscopy. The slides were then washed twice with cold ultrapure water to remove unbound proteins.

**Figure 10:**
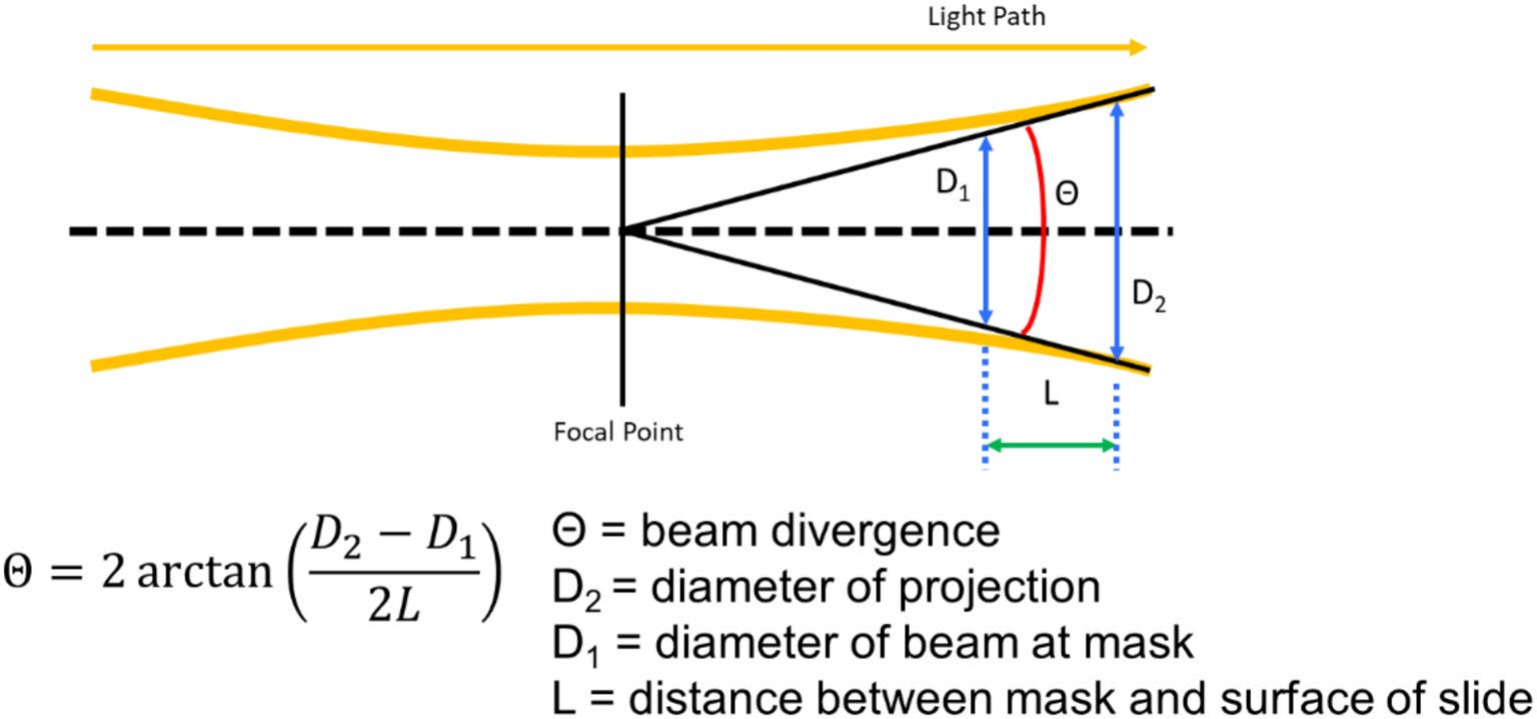
Schematic of photomask projection geometry.

To quantify the amount of mineralization within nylon photomask patterns, the projection of the mask onto the glass slide was calculated by measuring with digital calipers the distance between the top of the mask and the top of the glass L (1.1 ± 0.2 mm), determining D_1_ by imaging the photomask directly, and calculating D_2_ with the following relation (Fig. 10).

### PEGDA hydrogel formation and protein incorporation

Hydrogel disks (1-cm diameter) were formed via photopolymerization in glass and silicon molds. Prepolymer solutions were prepared by combining 0.1 g/mL 10 kDa PEGDA (10% w/v) with 10 mM PEG-RGDS, 1 ml HBS and 10 µL/mL photoinitiator solution (2,2-dimoxy-2-phenyl-acetophenone 300 mg/mL in N-vinyl-pyrrolidone; acetophenone/NVP). Separate solutions were supplemented with 200 ng/mL of a single type of PEGylated or non-PEGylated protein or peptide. The disks were formed by injecting the solution into 1 cm-diameter silicon molds clamped between glass slides and exposing the prepolymer molds to long-wavelength UV light (365 nm, 10 mW/cm^2^) for 1 min. The resulting gels were then washed in PBS to remove excess photoinitiator and allowed to swell in growth medium at 4 °C for 24 h before use.

### Cell culture

W-20-17 mouse bone marrow stromal cells (ATCC, Manassas, VA, USA) were seeded on PEGDA scaffolds at 10,000 cells/cm^2^. Cells were cultured in DMEM supplemented with 10% fetal bovine serum and 1% penicillin/streptomycin solution (10,000 Units penicillin and 10 mg streptomycin per mL). Seeded scaffolds were placed in 6 well transwells (Sigma Aldrich, St. Louis, MO, USA) and moved to new wells 12 h after seeding to retain only cells that attached to the surface. Media was replaced daily until differentiation analysis was conducted. After 4 days, the growth medium was aspirated from the wells. The gels were washed with PBS to remove any media residue. The cells were then stained for ALP activity using a Stemgent AP II staining kit, and the stain was visualized via light microscopy. The images were processed for stain intensity via ImageJ.

MC3T3-E1 mouse preosteoblast cells (ATCC, Manassas, VA, USA) were seeded on PEGDA scaffolds at 10,000 cells/cm^2^. Cells were cultured in MEM-α supplemented with 10% fetal bovine serum and 1% penicillin/streptomycin solution (10,000 Units penicillin and 10 mg streptomycin per mL). Seeded scaffolds were placed in 6 well transwells (Sigma Aldrich, St. Louis, MO, USA) and moved to new wells 12 h after seeding to retain only cells that attached to the surface. Media was replaced every other day until mineralization analysis was conducted. After 12 days, the growth medium was aspirated from the wells. The gels were washed with PBS to remove any media residue. The cells were fixed in 10% paraformaldehyde for 15 min, then stained for calcium mineralization via Alizarin red S (40 mM, pH 4.1). After removal of excess Alizarin red S solution and washing with ultrapure water, the cells were imaged using light microscopy. The images were processed for stain intensity via ImageJ.

MC3T3-E1 mouse preosteoblast cells were cultured on PEGDA hydrogels in the presence of PEGylated and non-PEGylated osteogenic proteins. After 12 days, the growth medium was aspirated from the wells. The gels were washed with PBS to remove any media residue. The cells were fixed in 10% paraformaldehyde for 15 min, then stained for calcium mineralization via Alizarin red S (40 mM, pH 4.1). After removal of excess Alizarin red S solution and washing with ultrapure water, the cells were imaged using light microscopy. The images were processed for stain intensity via ImageJ.

### Three-dimensional cell culture and mineralization

MC3T3-E1 mouse preosteoblast cells were encapsulated in PEGDA microspheres via water-in-oil emulsification followed by photopolymerization as previously described^22^. Briefly, A bulk prepolymer solution was prepared by dissolving 0.2 g poly(ethylene glycol) diacrylate, 0.30 µL triethanolamine, 0.20 µL eosin Y solution (1.0 mM), 0.2 µL pluronic acid, 7.5 µL photoinitiator solution (300 mg 2,2-dimethoxy-2-phenyl acetophenone in 1 mL 1-vinyl-2-pyrrolidinone) in 1 mL HBS (25 mM). The bulk prepolymer solution was then aliquoted into 10 separate vials. One growth factor was added to each vial to a final concentration of 200 ng/mL protein or PEG-protein equivalent. The prepolymer solutions were combined with an equal volume of cell suspension such that the final cell concentration was 5 × 10^6^ cells/mL. A separate mineral oil solution was prepared by adding 3 µL photoinitiator solution to 1 mL mineral oil. 100 µL of the cell/prepolymer solution was then added to 1 mL of the mineral oil solution in a borosilicate test tube and briefly vortexed to create and water-in-oil emulsion where the cells remained in the aqueous phase. The emulsion was then exposed to white light for 20 s with intermittent vortexing and rotation to maintain the integrity of the emulsion while evenly applying light to the entire tube.

The microsphere-encapsulated MC3T3-E1 cells were cultured in 12-well transwells (Sigma Aldrich, St. Louis, MO, USA) containing MEM-α supplemented with 10% fetal bovine serum and 1% penicillin/streptomycin solution (10,000 Units penicillin and 10 mg streptomycin per mL). Media was replaced every other day until mineralization analysis was conducted.

After 21 days, the microspheres were removed from the media, washed with ultrapure water, and fixed in 10% paraformaldehyde for 15 min. Each sample was then stained with Alizarin red S (40 mM, pH 4.1) for 15 min. After removal of excess Alizarin red S solution and washing with ultrapure water, the cells were imaged using light microscopy.

Mineralization was quantified within each image by assigning numeric value to the stain intensity of each microsphere within an image, adding up the values, and then dividing by the total number of microspheres within the image. Unstained microspheres were assigned a value of zero, partially-stained microspheres were assigned a value of 1, and fully stained microspheres were assigned a value of 2. Thus, the normalized mineralization value of an image ranges between 0 and 2.

### Patterning cellular osteogenic response

A 50 µL droplet of PEGylated protein solution (0.2 mg/mL) containing photoinitiator was pipetted onto the surface of each hydrogel and covered with a photomask transparency printed with a prescribed microscale pattern. The hydrogels were exposed to intense, white light passing through a focusing lens for 1 min to conjugate the PEGylated proteins to the hydrogel within the boundaries of the prescribed photomask pattern.

W-20-17 and MC3T3-E1 cells were cultured on the patterned hydrogel substrates as in Sect. 4.8. ALP activity in W-20-17 cells were visualized on day 4 via ALP staining using a Stemgent AP Staining Kit II. MC3T3-E1 cell monolayers were assayed for calcium mineralization after 12 days via Alizarin red S staining and imaged via brightfield color microscopy. The images were thresholded and converted to binary, thus isolating the stained cells, via ImageJ and checked against overlaid patterns. To check for patterned extracellular matrix, the images were passed through a color intensity threshold algorithm in Adobe Photoshop and checked against overlaid patterns.

## Supplementary information

Supplementary Figures.

## References

[1] Carragee EJ, Hurwitz EL, Weiner BK (2011). A critical review of recombinant human bone morphogenetic protein-2 trials in spinal surgery: Emerging safety concerns and lessons learned. Spine J..

[2] Giannoudis PV, Dinopoulos H, Tsiridis E (2005). Bone substitutes: An update. Injury.

[3] Marino JT, Ziran BH (2010). Use of solid and cancellous autologous bone graft for fractures and nonunions. Orthop. Clin. North Am..

[4] Mesfin A (2013). High-dose rhBMP-2 for adults: Major and minor complications: A study of 502 spine cases. J. Bone Joint Surg..

[5] Cahill KS, Chi JH, Day A, Claus EB (2009). Prevalence, complications, and hospital charges associated with use of bone-morphogenetic proteins in spinal fusion procedures. JAMA.

[6] Chen N-F (2010). Symptomatic ectopic bone formation after off-label use of recombinant human bone morphogenetic protein-2 in transforaminal lumbar interbody fusion. J. Neurosurg. Spine.

[7] Kang MH, Kim JS, Seo JE, Oh SC, Yoo YA (2010). BMP2 accelerates the motility and invasiveness of gastric cancer cells via activation of the phosphatidylinositol 3-kinase (PI3K)/Akt pathway. Exp. Cell Res..

[8] Lai T-H, Fong Y-C, Fu W-M, Yang R-S, Tang C-H (2008). Osteoblasts-derived BMP-2 enhances the motility of prostate cancer cells via activation of integrins. Prostate.

[9] Wong DA, Kumar A, Jatana S, Ghiselli G, Wong K (2008). Neurologic impairment from ectopic bone in the lumbar canal: A potential complication of off-label PLIF/TLIF use of bone morphogenetic protein-2 (BMP-2). Spine J..

[10] White KA, Olabisi RM (2019). Spatiotemporal control strategies for bone formation through tissue engineering and regenerative medicine approaches. Adv. Healthc. Mater..

[11] Baht GS, Hunter GK, Goldberg HA (2008). Bone sialoprotein–collagen interaction promotes hydroxyapatite nucleation. Matrix Biol..

[12] Lin S (2018). Tailored biomimetic hydrogel based on a photopolymerised DMP1/MCF/gelatin hybrid system for calvarial bone regeneration. J. Mater. Chem. B.

[13] Yu Y (2014). Dentin matrix proteins (DMPs) enhance differentiation of BMMSCs via ERK and P38 MAPK pathways. Cell Tissue Res..

[14] Schmoekel HG (2005). Bone repair with a form of BMP-2 engineered for incorporation into fibrin cell ingrowth matrices. Biotechnol. Bioeng..

[15] Sun J, Li J, Li C, Yu Y (2015). Role of bone morphogenetic protein-2 in osteogenic differentiation of mesenchymal stem cells. Mol. Med. Rep..

[16] Hunter GK, Goldberg HA (1993). Nucleation of hydroxyapatite by bone sialoprotein. Proc. Natl. Acad. Sci. USA.

[17] Rousseau M, Pereira-Mouriès L, Almeida M-J, Milet C, Lopez E (2003). The water-soluble matrix fraction from the nacre of Pinctada maxima produces earlier mineralization of MC3T3-E1 mouse pre-osteoblasts. Comp. Biochem. Physiol. B: Biochem. Mol. Biol..

[18] Wang J-J, Chen J-T, Yang C-L (2007). Effects of soluble matrix of nacre on bone morphogenetic protein-2 and Cbfa1 gene expressions in rabbit marrow mesenchymal stem cells. Nanfang Yike Daxue Xuebao.

[19] Metzler RA (2010). Nacre protein fragment templates lamellar aragonite growth. J. Am. Chem. Soc..

[20] Suzuki Y (2000). Alginate hydrogel linked with synthetic oligopeptide derived from BMP-2 allows ectopic osteoinduction in vivo. J. Biomed. Mater. Res..

[21] Peeters M (2015). BMP-2 and BMP-2/7 heterodimers conjugated to a fibrin/hyaluronic acid hydrogel in a large animal model of mild intervertebral disc degeneration. BioRes. Open Access.

[22] Olabisi RM (2010). Hydrogel microsphere encapsulation of a cell-based gene therapy system increases cell survival of injected cells, transgene expression, and bone volume in a model of heterotopic ossification. Tissue Eng. Part A.

[23] Liu H-W, Chen C-H, Tsai C-L, Lin IH, Hsiue G-H (2007). Heterobifunctional poly(ethylene glycol)-tethered bone morphogenetic protein-2-stimulated bone marrow mesenchymal stromal cell differentiation and osteogenesis. Tissue Eng..

[24] He X, Ma J, Jabbari E (2008). Effect of grafting RGD and BMP-2 protein-derived peptides to a hydrogel substrate on osteogenic differentiation of marrow stromal cells. Langmuir.

[25] Kolate A (2014). PEG—A versatile conjugating ligand for drugs and drug delivery systems. J. Control. Release.

[26] Leslie-Barbick JE, Shen C, Chen C, West JL (2011). Micron-scale spatially patterned, covalently immobilized vascular endothelial growth factor on hydrogels accelerates endothelial tubulogenesis and increases cellular angiogenic responses. Tissue Eng. Part A.

[27] Cooper GM (2010). Inkjet-based biopatterning of bone morphogenetic protein-2 to spatially control calvarial bone formation. Tissue Eng. Part A.

[28] Herberg S (2014). Inkjet-based biopatterning of SDF-1β augments BMP-2-induced repair of critical size calvarial bone defects in mice. Bone.

[29] Zouani OF, Chollet C, Guillotin B, Durrieu M-C (2010). Differentiation of pre-osteoblast cells on poly(ethylene terephthalate) grafted with RGD and/or BMPs mimetic peptides. Biomaterials.

[30] Chaturvedi R (2010). Isolation and Characterization of Major Bioactive Molecules from Nacre of Pinctada Fucata (Gould).

[31] R. M. Olabisi, M. R., C. L. Franco, J. Hoffmann & J. L. West. In *Biomedical Engineering Society Annual Meeting.*

[32] White, K., Franco, C., West, J. & Olabisi, R. In *Biomedical Engineering Society Annual Meeting.*

[33] He G, George A (2004). dentin matrix protein 1 immobilized on type I collagen fibrils facilitates apatite deposition in vitro. J. Biol. Chem..

[34] George, A., Guirado, E., & Chen, Y. DMP1 binds specifically to Type I collagen and regulates mineral nucleation and growth. In *Biomineralization* (pp. 137–145). Springer, Singapore (2018).

[35] Patel Z, Ueda H, Yamamoto M, Tabata Y, Mikos A (2008). In vitro and in vivo release of vascular endothelial growth factor from gelatin microparticles and biodegradable composite scaffolds. Pharm. Res..

[36] Patel ZS, Yamamoto M, Ueda H, Tabata Y, Mikos AG (2008). Biodegradable gelatin microparticles as delivery systems for the controlled release of bone morphogenetic protein-2. Acta Biomater..

[37] Patel ZS (2008). Dual delivery of an angiogenic and an osteogenic growth factor for bone regeneration in a critical size defect model. Bone.

[38] Gordon JAR (2007). Bone sialoprotein expression enhances osteoblast differentiation and matrix mineralization in vitro. Bone.

[39] Shen Y (2014). Engineering scaffolds integrated with calcium sulfate and oyster shell for enhanced bone tissue regeneration. ACS Appl. Mater. Interfaces.

