# Supplementary material for: Micropatterning biomineralization with immobilized mother of pearl proteins
Source: Sci Rep. 2021 Jan 25;11:2141. doi: 10.1038/s41598-021-81534-8 (PMC7835238; doi:10.1038/s41598-021-81534-8)
Supplement: Supplementary file 1 — Supplementary Figures. [file 41598_2021_81534_MOESM1_ESM.docx]

Supplementary Information for

**Micropatterning Biomineralization with Immobilized Mother of Pearl Proteins**

Kristopher A. White^1^, Vincent J. Cali^2^, Ronke M Olabisi^1^*

^1^Department of Biomedical Engineering, University of California—Irvine, Irvine, CA

^2^Department of Anatomy and Physiology, Queens College, City University of New York, Bayside, NY

*Correspondence should be addressed to RMO (ronke.olabisi@uci.edu)

- 1. Extraction of Nacre Water-soluble Matrix

After lyophilization, nacre WSM (0.072 g) was collected from powdered nacre (50 g). The dry protein was white in color and readily dissolved when reconstituted in aqueous medium.

- 1. Confirmation of PEGylation

Dentin matrix protein 1 (DMP1), BMP-2, BSP, the adhesion peptide RGDS, n16N and nacre WSM were successfully conjugated to PEG diacrylate (PEGDA). A ninhydrin assay of these PEGylated proteins indicated that between 10 and 20 percent conjugation of free amines were achieved for all protein samples. The conjugation efficiencies were observed to be 18.3 ± 5.1 percent, 15.4 ± 7.0 percent, 17.2 ± 7.7 percent, and 12.4 ± 9.7 percent for BMP-2, BSP, DMP1, and WSM, respectively (Figure S1). The conjugation efficiencies of the peptides were higher than that of the proteins, with efficiencies of 30.9 ± 2.9 and 90.1 ± 4.1 percent for n16N and RGDS, respectively. This is likely due to less steric interference as the molecular weight of n16N and RGDS are significantly lower than that of the full proteins, thus resulting in higher reaction efficiencies. Additionally, RGDS has only one primary amine at the N-terminal, resulting in a much more predictable reaction, with comparable efficiencies reported in the literature.^30^


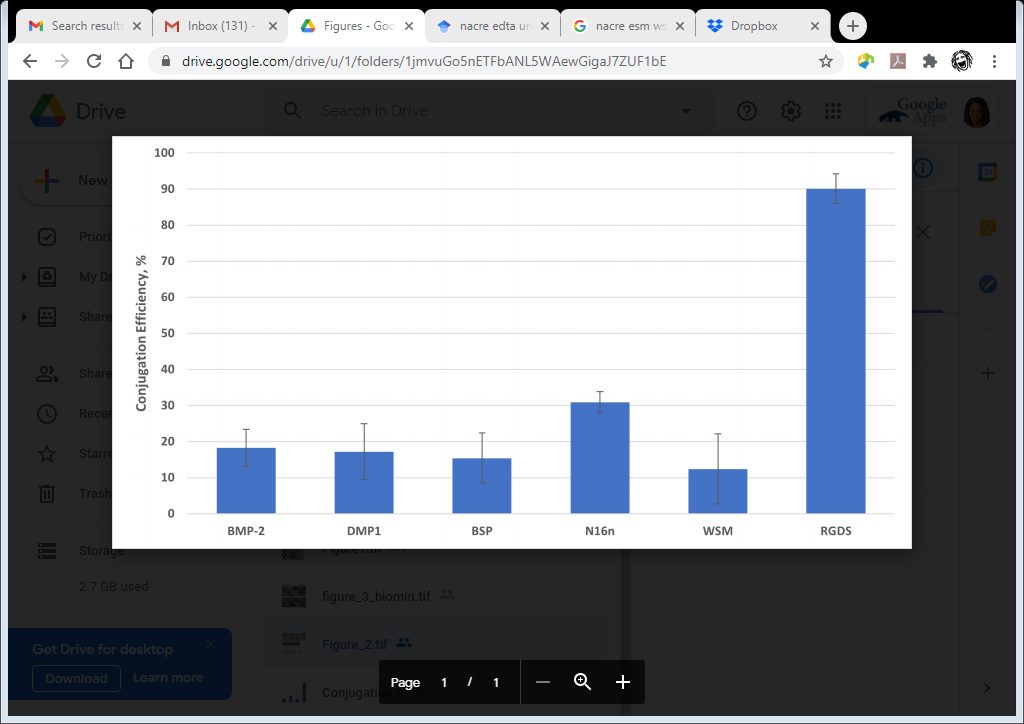


**Figure S1**: Conjugation efficiency of PEG-succinimidyl valerate (SVA) to free amines in each protein/peptide. Error bars represent standard deviation in triplicate samples.


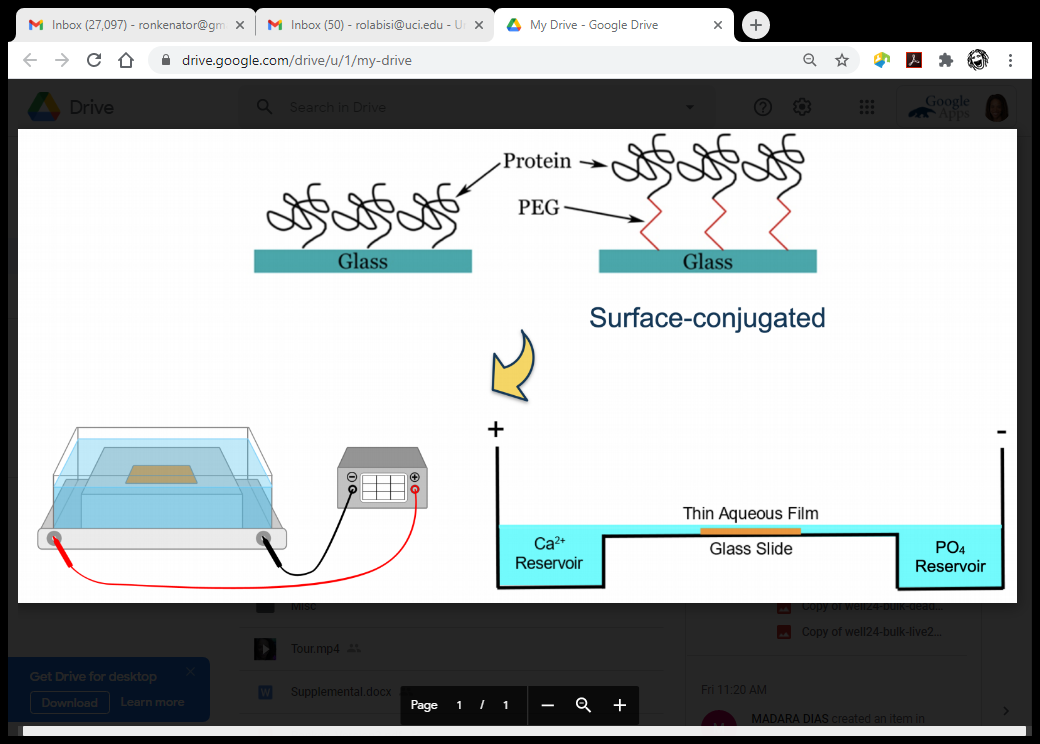


**Figure S2:** Acellular mineralization set-up for protein-adsorbed or surface conjugated proteins.

**
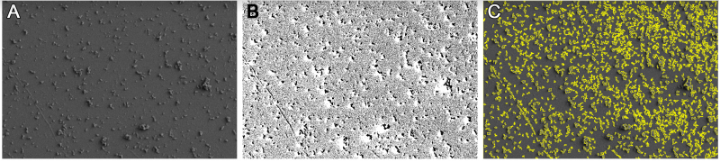
Figure S3**: ImageJ particle analysis on slides coated with PEG-WSM. **A:** The raw FE-SEM images **B:** were first thresholded to remove background features and color. **C:** Particles were then highlighted, counted, and characterized using the software’s particle analysis tool.

**
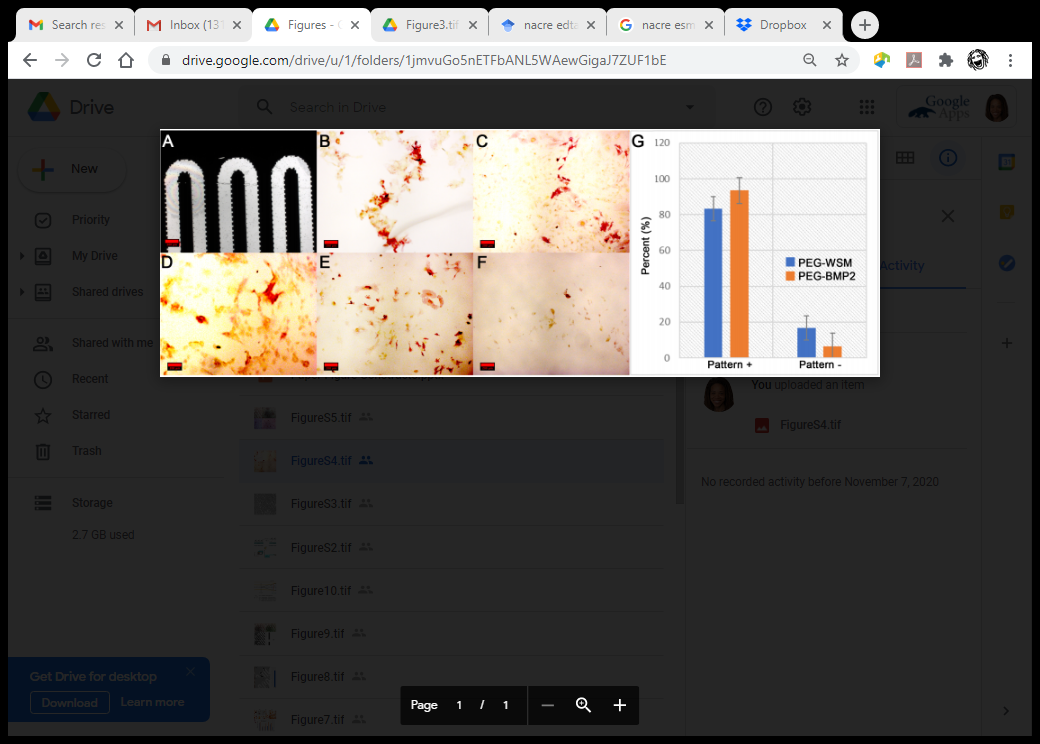
**

**Figure S4**: Alkaline phosphatase (ALP) staining of Day 4 W-20-17 cells seeded on PEG hydrogels patterned with PEGylated proteins. Red color indicates ALP activity. **A:** Hydrogels were patterned via photolithography through a transparency photomask pattern with **B:** nacre WSM proteins, **C:** BMP-2, **D:** DMP-1, **E:** BSP, and **F:** n16N. ALP activity appeared to be associated with patterns only in PEG-WSM and PEG-BMP-2 hydrogels (**G**). Scale bars are 200 µm. Error bars show standard deviation. Images were uniformly adjusted for brightness and contrast.


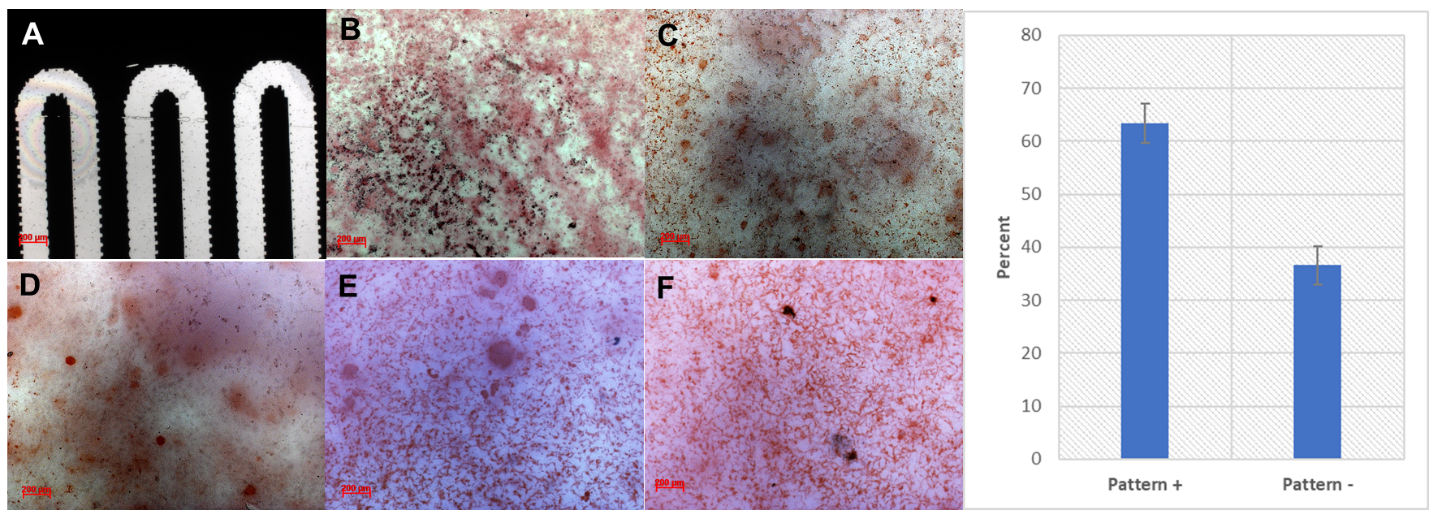

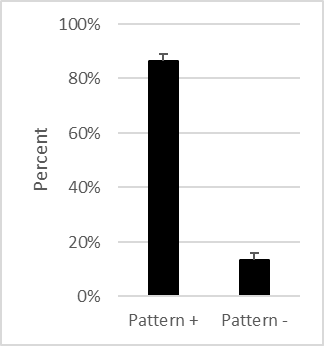


**Figure S5**: Alizarin red S stain of Day 12 MC3T3-E1 cells on patterned PEGDA hydrogels. **A:** Hydrogels were patterned via photolithography through a transparency photomask pattern with **B:** nacre WSM proteins, **C:** BMP-2, **D:** DMP-1, **E:** BSP, and **F:** n16N. Red stain is indicative of calcium mineralization. **B-F** show red stain, while only **B** shows red stain following a pattern. **G:** Red stain within or touching the pattern is quantified. Scale bars are 200 µm. Error bars show standard deviation.
